# *Trypanosoma cruzi* IV Causing Outbreaks of Acute Chagas Disease and Infections by Different Haplotypes in the Western Brazilian Amazonia

**DOI:** 10.1371/journal.pone.0041284

**Published:** 2012-07-25

**Authors:** Wuelton Marcelo Monteiro, Laylah Kelre Costa Magalhães, Amanda Regina Nichi de Sá, Mônica Lúcia Gomes, Max Jean de Ornelas Toledo, Lara Borges, Isa Pires, Jorge Augusto de Oliveira Guerra, Henrique Silveira, Maria das Graças Vale Barbosa

**Affiliations:** 1 Tropical Medicine Foundation Dr. Heitor Vieira Dourado, Manaus, Amazonas, Brazil; 2 University of the State of Amazonas, Manaus, Amazonas, Brazil; 3 Federal University of Amazonas, Manaus, Amazonas, Brazil; 4 State University of Maringá, Maringá, Paraná, Brazil; 5 Instituto de Higiene e Medicina Tropical, Center for Malaria Studies, Universidade Nova de Lisboa, Lisbon, Portugal; 6 Nilton Lins University Center, Manaus, Amazonas, Brazil; Universidade Federal de Minas Gerais, Brazil

## Abstract

**Background:**

Chagas disease is an emergent tropical disease in the Brazilian Amazon Region, with an increasing number of cases in recent decades. In this region, the sylvatic cycle of *Trypanosoma cruzi* transmission, which constitutes a reservoir of parasites that might be associated with specific molecular, epidemiological and clinical traits, has been little explored. The objective of this work is to genetically characterize stocks of *T. cruzi* from human cases, triatomines and reservoir mammals in the State of Amazonas, in the Western Brazilian Amazon.

**Methodology/Principal Findings:**

We analyzed 96 *T. cruzi* samples from four municipalities in distant locations of the State of Amazonas. Molecular characterization of isolated parasites from cultures in LIT medium or directly from vectors or whole human blood was performed by PCR of the non-transcribed spacer of the mini-exon and of the 24 S alfa ribosomal RNA gene, RFLP and sequencing of the mitochondrial cytochrome c oxidase subunit II (COII) gene, and by sequencing of the glucose-phosphate isomerase gene. The *T. cruzi* parasites from two outbreaks of acute disease were all typed as TcIV. One of the outbreaks was triggered by several haplotypes of the same DTU. TcIV also occurred in isolated cases and in *Rhodnius robustus*. Incongruence between mitochondrial and nuclear phylogenies is likely to be indicative of historical genetic exchange events resulting in mitochondrial introgression between TcIII and TcIV DTUs from Western Brazilian Amazon. TcI predominated among triatomines and was the unique DTU infecting marsupials.

**Conclusion/Significance:**

DTU TcIV, rarely associated with human Chagas disease in other areas of the Amazon basin, is the major strain responsible for the human infections in the Western Brazilian Amazon, occurring in outbreaks as single or mixed infections by different haplotypes.

## Introduction

Chagas disease is a parasitic disease caused by the flagellate protozoan *Trypanosoma cruzi*. The geographical distribution of sylvatic *T. cruzi* spreads from southern United States to southern Argentina and Chile, while domestic transmission is limited to Central and South America where domiciliated vector species occur [1], [2].

Despite the control of the Chagas disease in domestic and peridomestic cycles in the traditional transmission areas from Brazil, the infection is emerging as an important health problem in the Amazon Region of this country, with an increasing number of cases in recent decades [3], [4]. Information from the Brazilian Ministry of Health indicates that 756 cases of acute CD were reported in Brazil, from 2005 to October 2010. Strikingly of these 703 cases (93.0%) occurred in the Amazon Region [5]. Natural cycles of *T. cruzi* transmission are abundant and complex in the Amazon, involving a great diversity of wild mammal reservoirs and vectors, leading to intense infection rates of these hosts in the sylvatic environment [6]. The most frequent triatomine species are *Rhodnius pictipes*, *Rhodnius robustus* and *Panstrongylus geniculatus*. Under some circumstances, these sylvatic triatomines can invade houses, contaminate food, or attack forest workers [3]. Furthermore, a risk factor widely found in rural areas of this region is the building of houses close to palm tree woods occupied by triatomines and marsupials, both frequently infected by *T. cruzi* [4], [6]. In the Brazilian Amazon, prevalence of human *T. cruzi* infection may be estimated as 1–2%, but seems to be substantially higher in some subregions with relatively intense transmission, as in areas of *Leopoldinia piassava* palm trees in the Rio Negro, where the vector *Rhodnius brethesi* attacks workers during fiber collection activities [7]–[10].

Most of the acute cases of the disease registered in Amazon Region are associated with family outbreaks through oral transmission of the parasite [11], [12]. Scarce clinical descriptions of autochthonous acute cases demonstrate the predominance of nonspecific, usually prolonged, febrile illness [11]–[13]. Some studies show a more severe acute phase with the increased severity due to myocarditis [11], [12], [14], [15] and meningoencephalitis [16].

There appears to be geographical variation in the development of the clinical forms of the chronic disease in Latin America, but in the Brazilian Amazon only indeterminate and cardiac forms have been described [7]. Cross-sectional studies carried out in the Rio Negro showed a low severity profile for the chronic phase of the disease, attributed to the scarce parasitemia and/or to the lower pathogenicity of the sylvatic *T. cruzi* stocks circulating in this region [8], [9].

*T. cruzi* is genetically diverse and throughout the years independent genetic markers pointed to several evolutionary lineages. Most recently multilocus genotyping has consistently revealed six distinct ‘discrete typing units’ (DTUs), which have been divided into two ‘major subdivisions’ termed TcI and TcII; TcII being further split into five DTUs: TcIIa to TcIIe [17]. A recent consensus renamed these DTUs as TcIV, TcII, TcIII, TcV and TcVI, respectively [18]. TcII, TcV and TcVI predominate in the domestic transmission cycle in the South Cone of South America, where patients may present with severe acute disease or with chronic cardiac and/or digestive involvement [19], [20]. In the Brazilian Amazon, Venezuela, Colombia, Central and North America, TcI is the predominant DTU and the major cause of both acute and cardiac Chagas’ disease, but rare cases of “mega” syndromes [21]–[24] while TcIII and TcIV cause sporadic acute cases of Chagas’ disease in the Brazilian Amazon basin [25], [26].

Understanding the diversity of *T. cruzi* parasites circulating in the Amazon is important to the comprehension of the emergence and expansion of Chagas disease. Knowledge about molecular epidemiology of the emerging Chagas disease linked to the different *T. cruzi* lineages in the Amazon is essential for future control programs and in determining the relationship between specific molecular traits, parasite biology, and clinical and epidemiological patterns in this region. The purpose of this work, therefore, is to characterize the genetic types of *T. cruzi* from human cases, triatomines and reservoir mammals circulating in the State of Amazonas, Western Brazilian Amazon.

## Methods

### Ethics Statement

The study was approved by the Ethical Review Board of the Tropical Medicine Foundation of Amazonas (approval number 1940/08). Patients diagnosed with Chagas disease were treated according to the guidelines of the Brazilian Health Ministry. We obtained informed consent from all participants involved in our study.

### Area of Study

The State of Amazonas is located in the western North Region of Brazil (latitude 2°01′, longitude 73°48′), and comprises an area of 1,570,946.8 km^2^, with 62 municipalities. The estimated population of the state was 3,341,096 inhabitants in 2008, with 74.2% living in the urban zones and 25.8% in rural areas. Vegetation cover is mainly a dense evergreen rain forest. Climate is classified as the equatorial super-humid type, with rainfalls over 2,000 mm *per annum* and average annual temperatures between 26°C and 28°C. There is no clear distinction between dry and rainy seasons and the temperatures present a little variation throughout the state area.

### Parasites

We analyzed 96 samples from different hosts from four distant municipalities of Amazonas State. Forty six came from patients with acute Chagas disease and one was isolated from a chronic patient living in Manaus. Twenty seven stocks from acute cases were isolated in an outbreak in the municipality of Coari on April 2007 (AM21 to AM27) and fifteen samples from another outbreak which occurred in the municipality of Santa Isabel do Rio Negro on January 2010 (AM62 to AM69, AP60, Erlisson, D, Gus, L, LM, and W). Three stocks came from sole cases registered in the municipalities of Coari and one in Apuí. Following the protocol recommended by the Brazilian Ministry of Health, patients were treated with benznidazole, after the blood collection.

Thirty five samples were obtained from triatomines (*R. robustus* and *R. pictipes*) collected with Noireau traps [27] installed in palm trees in sylvatic and peridomestic environments, in the municipalities of Apuí, Coari, and Manaus. Fourteen samples were obtained from sylvatic reservoirs (*Didelphis albiventris* and *Philander opossum*) captured using Tomahawk traps with fruits as bait, in Manaus and Coari. Capture and handling for blood sample collection was performed according to permits from the Brazilian Institute for Environment (IBAMA) (approval number 1830651/07). The *T. cruzi* samples, hosts, method of isolation, and their geographical origins are shown in Supplementary Data (Table S1).

For *T. cruzi* isolation and culture, heparinized blood samples from humans were inoculated into tubes containing a biphasic medium consisting of NNN medium, covered with an overlay of LIT medium containing 10% fetal calf serum and 140 mg/ml of gentamycin sulphate [28]. Approximately 0.5 ml of whole blood was placed in each tube (3–5 tubes for each human/mammal). Cultures were kept at 28°C and monitored microscopically for parasite growth twice a week for two months. At the time of blood collection xenodiagnosis was also performed, using 20 third instar nymphs of *Triatoma infestans* or *Dipetalogaster maximus* per patient. The nymphs had not been fed for 60 days and were placed on the patients’ arms and left until feeding was considered complete. Nymphs were monitored at 30, 45 and 60 days after feeding, by abdominal compression and observation of the insect feces by microscopy, searching for trypomastigote forms. Positive triatomines were dissected and their intestinal contents were inoculated into the same medium used for hemocultures.

Field-collected triatomines were dissected and their intestinal contents were examined by phase microscopy. Positive samples for trypanosomes were cultured in NNN medium.

Isolates obtained in this study were cryopreserved in liquid nitrogen in the *T. cruzi* culture collection of the Tropical Medicine Foundation of Amazonas. When the parasites did not survive in culture, we used the strategy of genotyping the samples directly from patients’ blood or intestinal content of triatomines.

### *T. cruzi* DNA Extraction

After culturing *T. cruzi* parasites in LIT (liver infusion tryptose) medium containing 10% of inactivated fetal calf serum, at 28°C to reach a concentration of about 10^9^ cells/ml, extraction of total DNA from isolates was performed using the PureLink Kit (Invitrogen, Life technologies, USA), according to the manufacturer’s protocol. DNA was prepared from 200 µl of culture and eluted with 50 µl of milliQ water. For direct molecular characterization, DNA was extracted by the same procedure, using 200 µl of the triatomine intestinal content or whole human blood.

### Mini-exon Gene Analysis

DNA from the non-transcribed spacer of the mini-exon was amplified according to the multiplex protocol, as described previously [29]. Three oligonucleotides, derived from a hypervariable region of the *T. cruzi* mini-exon repeat and an oligonucleotide from a specific region of the *T. rangeli* non-transcribed spacer were used as upstream primers. A common downstream oligonucleotide was used as the opposing primer. After initial denaturing at 94°C for 1 minute, the samples were submitted to 35 cycles (94°C for 30 seconds more, 50°C for 30 seconds, and 72°C for 30 seconds), with a final extension at 72°C for 10 minutes. Amplified products were analyzed by agarose gel (3.0%) electrophoresis, and visualization with ultraviolet light after ethidium bromide staining. Amplicons of 150-bp are characteristic of TcIII/TcIV DTU, 200-bp of TcI and 250-bp of TcII, TcV and TcVI [30].

### Ribosomal RNA (rRNA) Gene Analysis

The 24 Sα rRNA gene sequence was amplified as described by Souto et al. [31] and revised by Macedo et al. [32]. After initial denaturing at 94°C for 1 minute, the samples were submitted to 30 cycles (94°C for 30 seconds more, 60°C for 30 seconds, and 72°C for 30 seconds), with a final extension at 72°C for 10 minutes. The amplified products were observed by silver staining in 6% polyacrylamide gel. Amplicons of 110-bp are characteristic of TcI/TcIII DTU, 125-bp of TcII/TcVI, 120 or 130-bp of TcIV and both 110 and 125 bp for TcV [31], [33], [34].

### Mitochondrial Cytochrome C Oxidase Subunit II (COII) Gene Analysis

The restriction fragment length polymorphism (RFLP) analysis for the COII gene was accessed using the methodology described by Freitas et al. [35] with modifications [36], using the primers Tcmit-10 and Tcmit-21, designed to amplify a ∼400 bp DNA fragment of the *T. cruzi* maxicircle. The amplification was processed with initial denaturing at 94°C for 1 minute and 30 cycles of 94°C for 30 seconds more, 48°C for 2 minutes, 72°C for 2 minutes, and final extension at 72°C for 10 minutes. Ten microlitres of the products of the *T. cruzi* DNA maxicircle was digested with 10 units of the restriction enzyme *AluI* (Invitrogen) for 16 hours. The RFLP analysis of the COII gene was done in 6.0% polyacrylamide gel and revealed by silver staining. At the polymorphic site *AluI*, the approximately 300-bp band is characteristic of *T*. *cruzi* I, the 250-bp band characteristic of *T. cruzi* II, and the bands larger than 300-bp characteristic of *T. cruzi* III to VI.

Sequencing of the COII gene was performed for 75 stocks as described previously [26]. The amplified PCR products were purified using SureClean Kit (Bioline, UK) and sequenced in both directions. PCR products were commercially sequenced by Macrogen (Korea).

### Glucose-phosphate Isomerase (GPI) Gene Analysis

A c.1 kb fragment of the GPI gene was amplified according to Gaunt et al. [37] using primers *gpi*.for (5′CGCACACTGGCCCTATTATT) and *gpi*.rev (5′TTCCATTGCTTTCCATGTCA) for a set of 63 stocks. The reaction cycle involved an initial denaturation step for five minutes at 94°C, followed by 28 amplification cycles (94°C for 30 seconds, 60°C for 30 seconds, 72°C for 30 seconds) and a final ten minutes elongation step at 72°C. PCR products were purified using SureClean Kit (Bioline, UK) and sequenced in both directions by Macrogen (Korea).

### Comparative Genetic Analysis

The band patterns obtained for *T. cruzi* samples obtained by miniexon, rRNA and COII/RFLP analyses were compared with the bands of reference samples of the six DTUs (TcI-TcVI), according to the Second Satellite Meeting [18]: Silvio X10 and CO1 17G2 (TcI), Esmeraldo and JG (TcII), 222 and 231 (TcIII), CAN III (TcIV), SO3 cl5 (TcV), and CL Brener (TcVI). Evolutionary relationships of the *Trypanosoma cruzi* samples sequenced were also performed by comparing them with clones belonging to known DTUs. The phylogenetic analysis of the GPI and COII sequences used the Neighbor-Joining method implemented in MEGA 4.1 [38]. The bootstrap consensus tree was inferred from 1500 replicates and the percentage of replicate trees in which the associated taxa clustered together are shown next to the branches. There were a total of 980 positions for GPI sequences and 400 positions for COII sequences in the final dataset. We defined haplotypes based on a set of single nucleotide polymorphisms in both GPI and COII sequences. The nucleotide sequences of COII gene (Accession numbers JN885398 to JN885445, GU178012 to GU178029, and JF322836) and of the GPI gene (Accession numbers JN885310 to JN885397) were deposited in GenBank.

## Results

### Mini-exon Gene Analysis

Of the 47 isolates from humans, 44 (93.6%) showed a 150-bp band of mini-exon compatible with TcIII/TcIV. These *T. cruzi* samples were isolated from two outbreaks occurred in the municipalities of Coari (AM01 to AM27) and Santa Isabel do Rio Negro (AM62 to AM69, AP060, D, Erlisson, GUS, L, LM, W), from a sole case from Coari (AM70), and from an isolated case registered in the municipality of Apuí (AM52). Three (6.4%) of the isolates from humans (AM36, AM49 and AM50) showed a 200-bp band compatible with TcI. Of the 35 isolates from triatomines, 31 (88.6%) were classified as TcI based on mini-exon analysis and 4 (11.4%) samples from the municipality of Apuí, all derived from *R. robustus*, showed the band pattern of TcIII/TcIV. All the 14 isolates from marsupials were typed as TcI using this marker. Mini-exon gene analysis was not able to distinguish TcIII and TcIV.

### Ribosomal RNA (rRNA) Gene Analysis

**Figure 1:**
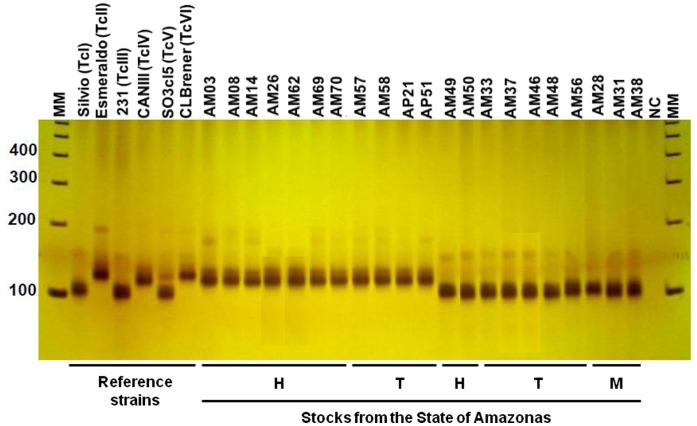
Representative polyacrylamide gel of the rRNA gene amplification for *Trypanosoma cruzi* isolates from humans (H), triatomines (T) and marsupials (M) from the State of Amazonas. MM, 100 bp molecular weight marker. NC, negative control.

Figure 1 shows that of the 47 isolates from humans, 44 (93.6%) showed 125-bp bands of rRNA compatible with TcIV consistently supporting their status as a DTU separate from TcIII. For human isolates AM36, AM49 and AM50, the rRNA analysis showed an approximately 110-bp band compatible with the TcI or TcIII DTUs. Analysis of rRNA was not able to distinguish TcI and TcIII. Four *R. robustus*-derived samples from the municipality of Apuí showed the band patterns of TcIV. The remaining samples from triatomines and all samples isolated from marsupials showed bands compatible with TcI or TcIII.

### Mitochondrial Cytochrome c Oxidase Subunit II (COII) Gene Analysis

**Figure 2:**
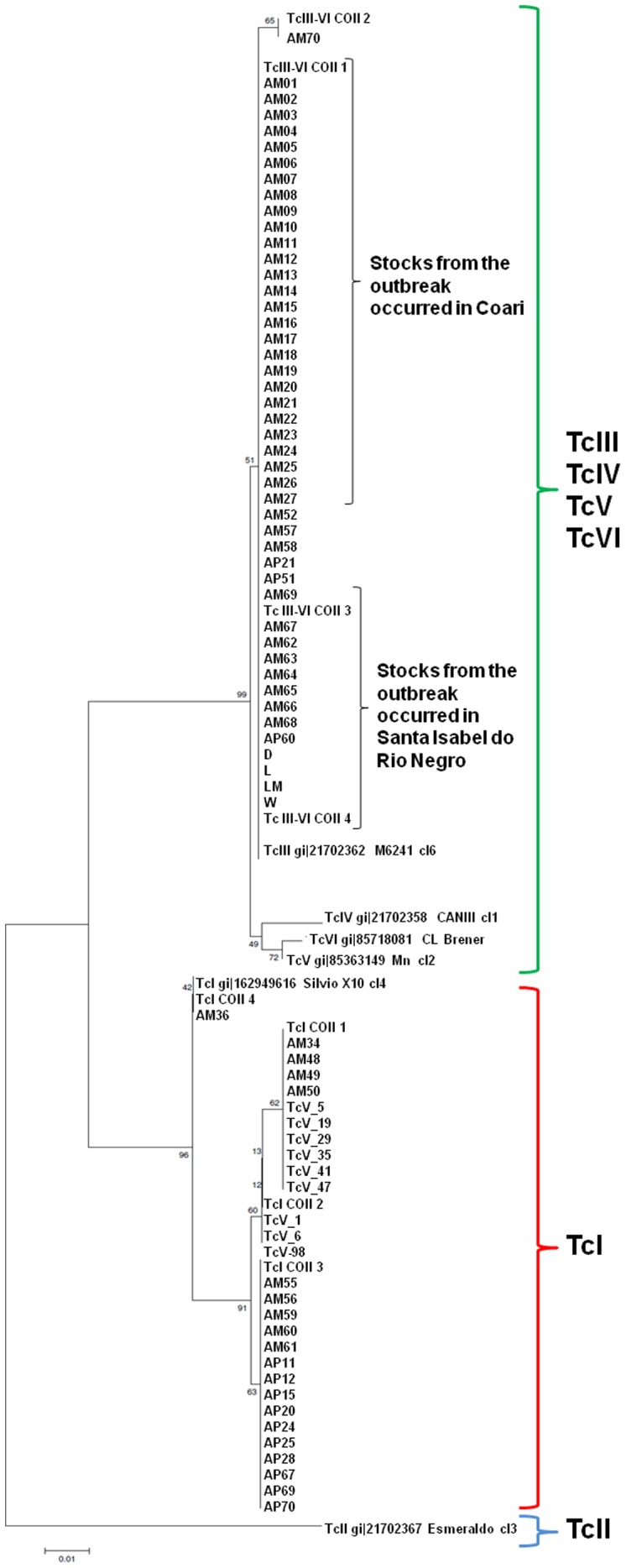
Allocation of the *Trypanosoma cruzi* isolated in the State of Amazonas into known phylogenetic clusters, most recently classified as TcI to TcVI, based on cytochrome c oxidase subunit II gene sequencing.

Bands larger than 300-bp in the COII/RFLP were found in 44 samples from humans and in four samples derived from *R. robustus*. Additionally, these samples showed COII sequences compatible with the third ancestral lineage according to Freitas et al. [35] (Figure 2). This set of samples and the reference strains of TcIII, TcIV, TcV and TcVI formed a single group that share a characteristic mitochondrial genome distinct from both TcI and TcII. The approximately 300-bp band characteristic of *T*. *cruzi* I was found in the remaining samples from triatomines and marsupials.

### Glucose-phosphate Isomerase (GPI) Gene Analysis

**Figure 3:**
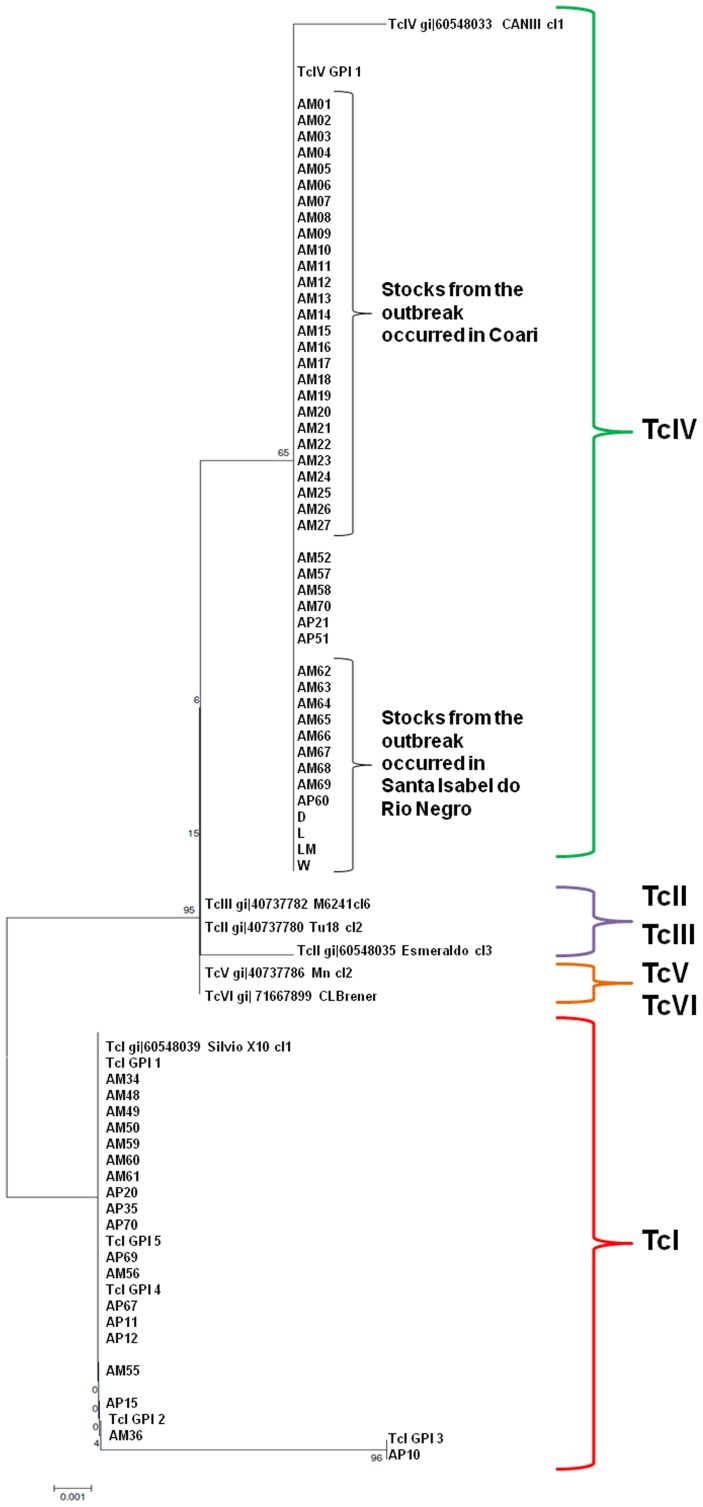
Allocation of the *Trypanosoma cruzi* isolated in the State of Amazonas into known phylogenetic clusters, most recently classified as TcI to TcVI, based on glucose-phosphate isomerase gene sequencing.

Data obtained from GPI sequencing showed that these isolates were more closely related to TcIV than with the other DTUs, although the not so high bootstrap values. GPI gene sequences compatible with TcIV were found for 44 samples from humans and for the four samples derived from *R. robustus*, in agreement with the results of the rRNA gene analysis (Figure 3). As per the other molecular markers, GPI gene sequencing has demonstrated similarity with TcI in the remaining samples from triatomines and marsupials.

The DTUs of the samples studied and the performance of the different molecular approaches were summarized in the Table S1 and in the Figure S1, respectively.

### Haplotypes

**Figure 4:**
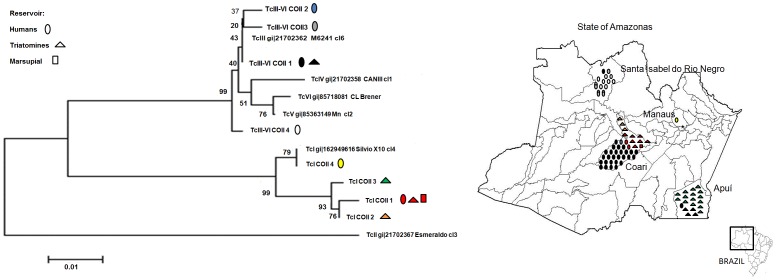
Geographic distribution and reservoirs of the haplotypes of the *Trypanosoma cruzi* isolated in the State of Amazonas, based on single-nucleotide polymorphisms identified by cytochrome c oxidase subunit II gene sequencing.

COII sequences analysis indicates the presence of four haplotypes of TcIV (TcIII-VI-COII-1 to 4) and two haplotypes of TcI (TcI-COII-1 and TcI-COII-4) circulating in humans; one haplotype of TcIV (TcIII-VI-COII-1) and three haplotypes of TcI (TcI-COII-1, TcI-COII-2, and TcI-COII-3) in triatomines; and one haplotype of TcI (TcI-COII-1) in the marsupial *P. opossum* (Figure 4).

**Figure 5:**
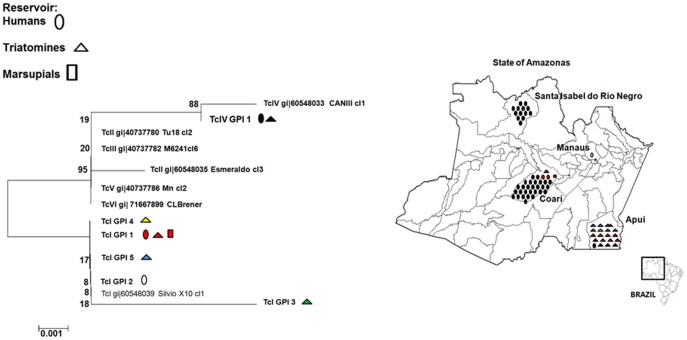
Geographic distribution and reservoirs of the haplotypes of the *Trypanosoma cruzi* isolated in the State of Amazonas, based on single-nucleotide polymorphisms identified by glucose-phosphate isomerase gene sequencing.

GPI sequencing showed one haplotype (TcIV-GPI-1) of TcIV circulating in humans and vectors; two haplotypes of TcI (TcI-GPI-1 and TcI-GPI-2) circulating in humans; four haplotypes of TcI (TcI-GPI-1, TcI-GPI-3, TcI-GPI-4, and TcI-GPI-5) harboured by triatomines; and one haplotype of TcI (TcI-GPI-1) in *P. opossum* (Figure 5).

Based on COII sequences obtained for human samples, haplotype TcIII-VI-COII-1 was the most widely distributed, occurring in all human acute cases from the outbreak in Coari, in the isolated case from Apuí (AM52) and together with other haplotypes in the outbreak of Santa Isabel do Rio Negro (AM69). Haplotype TcIII-VI-COII-2 was observed in an isolated case from Coari (AM70). Haplotypes TcIII-VI-COII-3 and TcIII-VI-COII-4 occurred in the outbreak of Santa Isabel do Rio Negro. The samples AM66, LM, and W, also from the outbreak of Santa Isabel do Rio Negro, presented a mixed population consisting of haplotypes TcIII-VI COII 3 and TcIII-VI-COII-4. GPI sequences were identical for all samples from humans typed as TcIV, constituting a single haplotype TcIV-GPI-1.

The isolate AM36, the only one cultured from a non-authochthonous chronic case, showed a COII gene sequence identical to the reference strain TcI Silvio X10, isolated from an acute human case in the state of Pará, in the Eastern Amazon region.

Single base mutation, identified by direct sequencing of the COII gene for TcI samples from triatomines, showed the presence of three haplotypes. All triatomines collected in Apuí, both *R. pictipes* and *R. robustus*, harboured the haplotype TcI-COII-3. Triatomines collected in Coari presented predominantly the haplotype TcI-COII-1, harboured by *R. pictipes* and *R. robustus*. Three specimens of *R. pictipes* from Coari presented the haplotype TcI-COII-2. GPI gene sequencing demonstrated only the haplotype TcI-GPI-1 in triatomines from Coari. In the municipality of Apuí, by other hand, four haplotypes were described in triatomines, predominantly TcI-GPI-1, followed by TcI-GPI-5, TcI-GPI-3 and TcI-GPI-4. The sample AP20 presented mixed infection by TcI-GPI-1 and TcI-GPI-5 haplotypes.

Four *R. robustus*-derived samples from the municipality of Apuí typed as TcIV (AM57, AM58, AP21, and AP51) belong to the haplotypes TcIII-VI-COII-1 and TcIV-GPI-1, according to COII and GPI sequences, respectively.

Sequencing techniques were performed only on one isolate from a specimen of *P. opossum* trapped in Coari, demonstrating the presence of the haplotypes TcI-COII-1 and TcI-GPI-1, according the COII and GPI sequences, respectively.

## Discussion

*T. cruzi* is a heterogeneous taxon with multiple hosts, vectors and routes of infection (for a review, see [39]). In the Brazilian Amazon region human cases of Chagas disease have been increasing due to uncontrolled migration and deforestation [3]. In this work, we showed that the majority of human acute cases in the state of Amazonas were from outbreaks, as observed previously in other Amazonian areas. We demonstrated that both TcI and TcIV were circulating in the same regions and were able to infect humans, resulting in acute infections. Previous studies described outbreaks of acute Chagas disease in the Amazon region that were dependent of TcI type [6], [12]. In our work, however, all isolates from the outbreaks were typed as TcIV even if they originated in two municipalities hundreds of km apart (Coari, in the Solimões River banks, and Santa Isabel do Rio Negro), thus indicating the emergence of a new epidemiologic profile in Brazil.

Interesting, COII analysis showed that all *T. cruzi* samples classified as TcIV based on nuclear gene markers presented mitochondrial gene products compatible with the third major lineage proposed by Freitas et al. [35]. COII sequences were less polymorphic and clustered together with the standard strains belonging to TcIII, TcIV, TcV, and TcVI. These findings are extremely important and confirm that TcIII and TcIV stocks from Amazon are not easily distinguished based on mitochondrial genes, as seen previously [40]. The phylogenetic status of these two DTUs, defined as Z3 in the Miles’s original classification [41], is still a matter for debate [35], [40]. DTU TcIII corresponds to Z3A and TcIIc strains, while DTU TcIV corresponds to Z3B and TcIIa according to Zingales et al. [18]. Based on mosaic patterns of nucleotide diversity across nine nuclear genes, Westenberger et al. [42] proposed that both are the product of an early hybridization between lineages TcI and TcII. Others argue that TcIII and TcIV represent a single ancestral group in their own right, because these lineages share a characteristic mitochondrial genome [35] and chromosome size variation [43] dissimilar from both TcI and TcII.

Analysis of both COII [40] and CYTB [44] shows far less mitochondrial diversity both within and between TcIII and TcIV (from South America) than would be expected in light of the divergence observed for slower-evolving nuclear genes. This implies a mechanism acting to homogenize maxicircle sequences while nuclear sequences remain free to diverge. Our work includes TcIV from Western Brazilian Amazon in these clade based on COII analysis, but our findings agree that nuclear gene sequences consistently support their status as a genetically separate clade [40], [42], [44]–[47] consistent with different size phenotypes of TcIII and TcIV observed by flow cytometry analysis [48].

There are few reports concerning the ecogeographical and epidemiological traits of TcIV, undermining any inference about the way that the aforementioned outbreaks were triggered. Sylvatic hosts of TcIV are not conclusively known in the Amazon Region. Despite previous records in *Monodelphis*, *Dasypus*, primates, and *Panstrongylus* from molecular analyses [49]–[52], and although human isolates were all identified by robust molecular markers, only seven human isolates (including CANIII and JJ) were confirmed as TcIV prior to this work [51]. Moreover, vectors infected with this lineage have also been poorly characterized, except *R. brethesi*, which is restricted to Northern Amazonia [29], [43], [50], [51], [53], and *R. robustus*, a widely distributed species [51]. Here, we corroborate that *R. robustus* harbours TcIV, and is circulating in an arboreal transmission cycle in sympatry with TcI and is distinct from the terrestrial ecotopes usually attributed to TcIII [51]. We could not observe TcIV in the *R. pictipes* collected. TcIV was not found in didelphids in this study, in line with other studies [50], [51]. Our observation of this DTU in distinct areas extends its known range in the vast Amazon basin. The limited data about *T. cruzi* DTUs in wild reservoirs and triatomines in the Amazon is insufficient to rule out other arboreal or even terrestrial mammals and vectors as natural hosts of TcIV.

One may speculate that the low parasitemia and morbidity of the chronic Chagas disease in this state (despite a high seroprevalence in some areas [3], [7]), contrasting with the florid clinical manifestations in the acute phase of the disease [11], [12], [14]–[16], is due to the type of parasites circulating. Acquiring knowledge about the biological properties of the TcIV lineage, which certainly plays an important role in the Chagas disease pathogenesis and response to the specific chemotherapy in the Amazon region, represents a challenge for future research. Our preliminary results indicate that Amazonian *T. cruzi* isolates promote scarce parasitemia and low virulence and pathogenicity in mice in comparison to TcII strains [54], [55]. At least in Brazil, TcII and rarely TcI, appear to be exclusively responsible for the tissue lesions in chronic Chagas disease [19], [23], [56], [57]. However, chronic disease (cardiac-digestive form) has been reported for Z3 type parasites that clustered with CANIII (reference strain of the DTU TcIV) in the multilocus enzyme electrophoresis analysis [58], which highlights the need to characterize the epidemiological and clinical features associated with the different DTUs of the emergent Chagas disease in the Amazon.

Patients belonging to the outbreak occurred in Coari had acute febrile illness accompanied by headache, myalgia, epigastric pain, vomiting and oedema of the face and lower limbs [26]. The most frequent signs and symptoms in the outbreak of Santa Isabel do Rio Negro were fever, asthenia, abdominal pain, palpitations, diarrhea and generalized or facial oedema [59]. No severe cases or deaths were registered among these patients. Preliminary investigations showed suppression of the parasitemia and symptoms after starting treatment with benznidazole.

The isolation of *T. cruzi* stocks with identical genetic sequences suggests a single source of infection for the outbreak occurred in the municipality of Coari. However, in the outbreak from Santa Isabel do Rio Negro, three haplotypes were registered based on COII sequencing and, interestingly, three patients presented two haplotypes simultaneously. Strikingly, our group observed differences between the isolates from the outbreaks in Coari and Santa Isabel do Rio Negro regarding parasitological parameters in mice, suggesting higher virulence for the last [54]. This finding suggests superinfection from discrete sources as well as the simultaneous transmission of multiclonal parasite populations by a single triatomine. *T. cruzi* hosts and vectors have occasionally been identified with mixed infections of different DTUs [36], [60]–[63] and preliminary data demonstrate that multiple variants of the same DTU could also be present [64]–[66]. Analysis of *T. cruzi* isolates from an acute Chagas disease outbreak in the State of Pará human showed TcI and Z3 as concurrent causative agents [12]. This study confirmed the wealth of parasite genetic diversity that can exist in an outbreak likely to have been caused by oral transmission, and looked at, for the first time, subpopulations amongst the affected patients.

The geographical area from which we have taken *T. cruzi* stocks, although vast, is only a fraction of the Amazonian area. Given the natural species distribution in its biomes there is no doubt that the full range of genetic lineage diversity within the *T. cruzi* isolates is more complex. In the Amazon basin, expansion of human populations into previously undisturbed cycles of natural transmission of *T. cruzi* may contribute to transmission of Chagas disease by the accidental introduction of wild vectors harboring TcI and TcIV into human food chain or by contact of these vectors with humans because of environmental changes. This is the probable mechanism explaining the emergence of this genotype in this region, evidenced by its predominance in our study. Characterization is needed of *T. cruzi* from more autochthonous chronic cases from the Amazon region, particularly the re-isolation and re-examination of strains from acute cases produced by TcI and TcIV lineages.

In summary, the lineages TcIV and TcI overlap in the Western Amazon region. The first DTU predominated among human cases, being responsible for triggering two impressive outbreaks caused by probable oral transmission. There were instances of the sharing of identical or nearly identical mitochondrial haplotypes between TcIV strains and strains from other DTUs (TcIII, TcV or TcVI) for which nuclear GPI sequences were divergent. Such incongruence between mitochondrial and nuclear phylogenies is likely indicative of historical genetic exchange events resulting in mitochondrial introgression between DTUs. Furthermore, we confirmed that outbreaks by this DTU can be due to single or mixed haplotypes. Studies into the complexity of mixed infections within an individual host need to be carried out. Due to the few data on the relationship between infra-DTU genotypes/haplotypes and clinico-epidemiological features of Chagas disease and parasite-vector interaction, we highlight the need of future studies focusing on this subjects. These results will help to clarify the peculiarities of Chagas disease epidemiology in the Amazonia.

## Supporting Information

Figure S1 **Summary of the different molecular approaches used to determine the DTUs of the samples studied.**
(PPTX)

Table S1 **Geographic origin, host, isolation method, and discrete typing units (DTUs) of** ***Trypanosoma cruzi*** **stocks from the State of Amazonas used in the study.**
(DOCX)
