# Supplementary material for: Trypanosoma cruzi IV Causing Outbreaks of Acute Chagas Disease and Infections by Different Haplotypes in the Western Brazilian Amazonia
Source: PLoS One. 2012 Jul 25;7(7):e41284. doi: 10.1371/journal.pone.0041284 (PMC3405119; doi:10.1371/journal.pone.0041284)
Supplement: Table S1 — Geographic origin, host, isolation method, and discrete typing units (DTUs) of Trypanosoma cruzi stocks from the State of Amazonas used in the study. (DOCX) [file pone.0041284.s002.docx]

Supporting Information. Geographical origins, hosts, isolation method, and discrete typing units (DTUs) of *Trypanosoma cruzi* stocks from the State of Amazonas.

| **Code** | **Geographic origin** | **Host** | **Isolation method** | **DTU** |
| --- | --- | --- | --- | --- |
| **AM01** | Coari | *Homo sapiens* | Hemoculture | TcIV |
| **AM02** | Coari | *Homo sapiens* | Hemoculture | TcIV |
| **AM03** | Coari | *Homo sapiens* | Hemoculture | TcIV |
| **AM04** | Coari | *Homo sapiens* | Hemoculture | TcIV |
| **AM05** | Coari | *Homo sapiens* | Hemoculture | TcIV |
| **AM06** | Coari | *Homo sapiens* | Hemoculture | TcIV |
| **AM07** | Coari | *Homo sapiens* | Hemoculture | TcIV |
| **AM08** | Coari | *Homo sapiens* | Hemoculture | TcIV |
| **AM09** | Coari | *Homo sapiens* | Hemoculture | TcIV |
| **AM10** | Coari | *Homo sapiens* | Hemoculture | TcIV |
| **AM11** | Coari | *Homo sapiens* | Hemoculture | TcIV |
| **AM12** | Coari | *Homo sapiens* | Hemoculture | TcIV |
| **AM13** | Coari | *Homo sapiens* | Hemoculture | TcIV |
| **AM14** | Coari | *Homo sapiens* | Hemoculture | TcIV |
| **AM15** | Coari | *Homo sapiens* | Hemoculture | TcIV |
| **AM16** | Coari | *Homo sapiens* | Hemoculture | TcIV |
| **AM17** | Coari | *Homo sapiens* | Hemoculture | TcIV |
| **AM18** | Coari | *Homo sapiens* | Hemoculture | TcIV |
| **AM19** | Coari | *Homo sapiens* | Xenodiagnosis | TcIV |
| **AM20** | Coari | *Homo sapiens* | Xenodiagnosis | TcIV |
| **AM21** | Coari | *Homo sapiens* | Xenodiagnosis | TcIV |
| **AM22** | Coari | *Homo sapiens* | Xenodiagnosis | TcIV |
| **AM23** | Coari | *Homo sapiens* | Xenodiagnosis | TcIV |
| **AM24** | Coari | *Homo sapiens* | Xenodiagnosis | TcIV |
| **AM25** | Coari | *Homo sapiens* | Xenodiagnosis | TcIV |
| **AM26** | Coari | *Homo sapiens* | Xenodiagnosis | TcIV |
| **AM27** | Coari | *Homo sapiens* | Xenodiagnosis | TcIV |
| **AM28** | Manaus | *Didelphis marsupialis* | Hemoculture | TcI |
| **AM29** | Manaus | *Didelphis marsupialis* | Hemoculture | TcI |
| **AM30** | Manaus | *Didelphis marsupialis* | Xenodiagnosis | TcI |
| **AM31** | Manaus | *Didelphis marsupialis* | Xenodiagnosis | TcI |
| **AM32** | Manaus | *Didelphis marsupialis* | Hemoculture | TcI |
| **AM33** | Manaus | *Rhodnius pictipes* | Xenoculture | TcI |
| **AM34** | Coari | *Philander opossum* | Hemoculture | TcI |
| **AM35** | Manaus | *Didelphis marsupialis* | Hemoculture | TcI |
| **AM36** | Manaus | *Homo sapiens* ^(a)^ | Xenodiagnosis | TcI |
| **AM37** | Coari | *Rhodnius robustus* | Xenoculture | TcI |
| **AM38** | Coari | *Philander opossum* | Hemoculture | TcI |
| **AM39** | Manaus | *Didelphis marsupialis* | Hemoculture | TcI |
| **AM40** | Manaus | *Didelphis marsupialis* | Xenodiagnosis | TcI |
| **AM42** | Manaus | *Didelphis marsupialis* | Hemoculture | TcI |
| **AM43** | Manaus | *Didelphis marsupialis* | Hemoculture | TcI |
| **AM44** | Manaus | *Didelphis marsupialis* | Hemoculture | TcI |
| **AM45** | Manaus | *Didelphis marsupialis* | Hemoculture | TcI |
| **AM46** | Manaus | *Rhodnius robustus* | Not performed | TcI |
| **AM47** | Manaus | *Rhodnius pictipes* | Not performed | TcI |
| **AM48** | Coari | *Rhodnius pictipes* | Xenoculture | TcI |
| **AM49** | Coari | *Homo sapiens* | Hemoculture | TcI |
| **AM50** | Coari | *Homo sapiens* | CSF ^(b)^ culture | TcI |
| **AM52** | Apuí | *Homo sapiens* | Hemoculture | TcIV |
| **AM55** | Apuí | *Rhodnius robustus* | Inoculation in mice | TcI |
| **AM56** | Apuí | *Rhodnius robustus* | Inoculation in mice | TcI |
| **AM57** | Apuí | *Rhodnius robustus* | Inoculation in mice | TcIV |
| **AM58** | Apuí | *Rhodnius robustus* | Inoculation in mice | TcIV |
| **AM59** | Apuí | *Rhodnius pictipes* | Inoculation in mice | TcI |
| **AM60** | Apuí | *Rhodnius pictipes* | Inoculation in mice | TcI |
| **AM61** | Apuí | *Rhodnius pictipes* | Inoculation in mice | TcI |
| **AM62** | Santa Isabel do Rio Negro | *Homo sapiens* | Hemoculture | TcIV |
| **AM63** | Santa Isabel do Rio Negro | *Homo sapiens* | Hemoculture | TcIV |
| **AM64** | Santa Isabel do Rio Negro | *Homo sapiens* | Hemoculture | TcIV |
| **AM65** | Santa Isabel do Rio Negro | *Homo sapiens* | Hemoculture | TcIV |
| **AM66** | Santa Isabel do Rio Negro | *Homo sapiens* | Hemoculture | TcIV |
| **AM67** | Santa Isabel do Rio Negro | *Homo sapiens* | Hemoculture | TcIV |
| **AM68** | Santa Isabel do Rio Negro | *Homo sapiens* | Hemoculture | TcIV |
| **AM69** | Santa Isabel do Rio Negro | *Homo sapiens* | Hemoculture | TcIV |
| **AP60** | Santa Isabel do Rio Negro | *Homo sapiens* | Xenodiagnosis | TcIV |
| **Erlisson** | Santa Isabel do Rio Negro | *Homo sapiens* | Xenodiagnosis | TcIV |
| **D** | Santa Isabel do Rio Negro | *Homo sapiens* | Not performed | TcIV |
| **Gus** | Santa Isabel do Rio Negro | *Homo sapiens* | Not performed | TcIV |
| **L** | Santa Isabel do Rio Negro | *Homo sapiens* | Not performed | TcIV |
| **LM** | Santa Isabel do Rio Negro | *Homo sapiens* | Not performed | TcIV |
| **W** | Santa Isabel do Rio Negro | *Homo sapiens* | Not performed | TcIV |
| **AM70** | Coari | *Homo sapiens* | Hemoculture | TcIV |
| **AP10** | Apuí | *Rhodnius robustus* | Not performed | TcI |
| **AP11** | Apuí | *Rhodnius pictipes* | Not performed | TcI |
| **AP12** | Apuí | *Rhodnius robustus* | Not performed | TcI |
| **AP15** | Apuí | *Rhodnius pictipes* | Not performed | TcI |
| **AP20** | Apuí | *Rhodnius robustus* | Not performed | TcI |
| **AP21** | Apuí | *Rhodnius robustus* | Not performed | TcIV |
| **AP24** | Apuí | *Rhodnius pictipes* | Not performed | TcI |
| **AP25** | Apuí | *Rhodnius robustus* | Not performed | TcI |
| **AP28** | Apuí | *Rhodnius robustus* | Not performed | TcI |
| **AP35** | Apuí | *Rhodnius pictipes* | Not performed | TcI |
| **AP51** | Apuí | *Rhodnius robustus* | Not performed | TcIV |
| **AP67** | Apuí | *Rhodnius robustus* | Not performed | TcI |
| **AP69** | Apuí | *Rhodnius robustus* | Not performed | TcI |
| **AP70** | Apuí | *Rhodnius robustus* | Not performed | TcI |
| **TcV_1** | Coari | *Rhodnius pictipes* | Not performed | TcI |
| **TcV_5** | Coari | *Rhodnius pictipes* | Not performed | TcI |
| **TcV_6** | Coari | *Rhodnius pictipes* | Not performed | TcI |
| **TcV_19** | Coari | *Rhodnius robustus* | Not performed | TcI |
| **TcV_29** | Coari | *Rhodnius robustus* | Not performed | TcI |
| **TcV_35** | Coari | *Rhodnius pictipes* | Not performed | TcI |
| **TcV_41** | Coari | *Rhodnius pictipes* | Not performed | TcI |
| **TcV_47** | Coari | *Rhodnius pictipes* | Not performed | TcI |
| **TcV_98** | Coari | *Rhodnius pictipes* | Not performed | TcI |

a: The only stock from a human in the chronic phase; b: Cerebrospinal fluid.
